# Propagation Speed of Dynamic Mode-I Cracks in Self-Compacting Steel Fiber-Reinforced Concrete

**DOI:** 10.3390/ma13184053

**Published:** 2020-09-12

**Authors:** Kaiming Pan, Rena C. Yu, Xiaoxin Zhang, Gonzalo Ruiz, Zhimin Wu

**Affiliations:** 1 Escuela Técnica Superior de Ingenieros de Caminos, Canales y Puertos, University of Castilla-La Mancha, 13071 Ciudad Real, Spain; Kaiming.Pan@alu.uclm.es (K.P.); Gonzalo.Ruiz@uclm.es (G.R.); 2 Escuela de Ingeniería Minera e Industrial de Almadén, University of Castilla-La Mancha, 13400 Ciudad Real, Spain; Xiaoxin.Zhang@uclm.es; 3 Institute of Structural Engineering, Faculty of Infrastructure Engineering, Dalian University of Technology, Dalian 116024, China; wuzhimin@dlut.edu.cn

**Keywords:** SFRSCC, crack propagation velocity, Digital Image Correlation (DIC), crack initiation strain rate, the terminal crack velocity

## Abstract

The objective of this study is to measure the crack propagation speed in three types of self-compacting concrete reinforced with steel fibers loaded under four different loading rates. Central-notched prismatic beams with two types of fibers (13 mm and 30 mm in length), three fiber volume ratios, 0.51%, 0.77% and 1.23%, were fabricated. Four strain gages were glued on one side of the specimen notch to measure the crack propagation velocity, a fifth one at the notch tip to estimate the strain rates upon the initiation of a cohesive crack and the stress-free crack. A servo-hydraulic testing machine and a drop-weight impact device were employed to conduct three-point bending tests at four loading-point displacement rates, the former to perform tests at 2.2 μm/s, 22 mm/s and the latter for those at 1.77 m/s, 2.66 m/s, respectively. With lower fiber contents, smooth mode-I cracks were formed, the crack speed reached the order of 1 mm/s and 20 m/s. However, crack velocities up to 1417 m/s were obtained for the concrete with high content of fibers under impact loading. This value is fairly close to the theoretically predicted terminal crack velocity of 1600–1700 m/s. Numerical simulations based on cohesive theories of fracture and preliminary results based on the technique of Digital Image Correlation are also presented to complement those obtained from the strain gages. In addition, the toughness indices are calculated under all four loading rates. Strain hardening (softening) behavior accounting from the initiation of the first crack is observed for all three types of concrete at low (high) loading rates. Significant enhancement in the energy absorption capacity is observed with increased fiber content.

## 1. Introduction

Crack propagation in concrete is the main mechanism of material failure and this process is often complex, especially under dynamic loading. Consequently, knowing at what speed and in which direction a crack can propagate under different loading conditions is of great importance. Different experimental techniques such as strain gages [1,2,3,4,5,6,7,8,9,10], acoustic emission [11] and digital image correlation (DIC) [12,13,14] have been employed to measure crack propagation velocity in concrete. The earliest measurement of crack velocity in plain concrete goes back to the work of Curbach and Eibl [1] in 1989, in which a silver barrier was spread on the expected crack path to measure its velocity in a notched beam impacted by a steel cylinder. Velocities up to 500 to 700 m/s were recorded. Regarding fiber-reinforced concrete (FRC), Mindess et al. [15] measured crack velocities of up to 190 m/s for Dramix-fiber concrete. Ngo et al. [13] and Pyo et al. [14] reported crack velocity of up to 984 m/s and 1454 m/s, respectively, in ultra-high performance fiber reinforced concrete (UHPFRCs) with DIC. Zhang et al. [3,6] used strain gages on beams impacted by a drop weight impact device and measured crack velocities up to 417 m/s in plain concrete and 690 m/s in FRC. Recently, through inspection of high-speed video recordings, Ruiz et al. [16] reported speed up to 650 m/s of mixed-mode crack in steel fiber-reinforced self-compacting concrete (SFRSCC). However, the effect of loading rate and fiber volume fraction on the crack propagation velocity in these relatively novel materials, is still in need of further investigation. In addition, the obtained maximum crack speeds are far less than the expected terminal crack velocities, which are estimated as up to 38% of the longitudinal wave velocity [17].

In this work, strain gages glued to the surface of central notched beams were used to measure the mode-I crack velocities and to detect the strain rate upon crack initiation at the notch tip. These beams were made from the same self-compacting concrete and reinforced with two types of steel fibers, the short one was 13 mm in length, 0.2 mm in diameter, the long one was 30 mm in length and 0.38 mm in diameter. Three concrete types were designed and fabricated with three different volume ratios, 0.51%, 0.77% and 1.23%. Tests at four loading rates, two of which achieved through a servo-hydraulic testing machine, the other two with a drop-weight impact device, were carried out. Preliminary results using the technique of Digital Image Correlation are also presented to contrast with the measurements obtained from the strain gages. In addition, numerical simulations based on the cohesive theories of fracture [18], were performed and the results are compared with the experimental measurements. The key novelties of the current work lie in the following aspects. First, a high speed of mode-I crack speed (1417 m/s), close to the terminal crack velocity and measured using strain gage readings was for the first time reported in the lab. Second, strain rates upon the initiation of both a cohesive crack and a stress-free crack are identified and for the first time measured. Third, the measured crack speed using strain gage method and the technique of DIC are complemented with numerical simulations. Fourth, the effect of fiber content on strain rate and crack propagation speed are explored.

The rest of the this paper is organized as follows. The experimental program is described next. The results and discussion including the numerical simulations are presented in Section 3. Finally, relevant conclusions are drawn in Section 4.

## 2. Experimental Program

The specimens were made from three types of self-compacting concrete. Different fiber types and fiber contents were added to the same base concrete. They were named as concrete PA, PB and PC for the fiber volume fractions of 0.51%, 0.77% and 1.23%, respectively. It needs to be pointed out that, the same material characterization and experimental tests, except the utilization of strain gages and DIC, were carried out in the work of Zhang et al. [19], therefore we only briefly recount the material characterization herein. The load and strain rate upon crack initiation, the toughness indices and residual strength factors, the crack propagation velocity measurement are subsequently described.

### 2.1. Material Characterization

The base concrete mix proportion is 1:0.12:0.35:1.12:1.27:0.38:0.021 (cement:silica fume:silica filler:fine sand:coarse sand:water:super-plasticizer). Specifically, CEM I cement 42.5 R-SR, two kinds of high-quality natural river sand (the maximum particle size was of 0.8 mm and 2 mm, respectively), two types of superplasticizers, Glenium ACE-325 and B-225, were used. Strictly speaking, since the maximum size of aggregate is less than 8 mm, and the compressive strength is above 100 MPa, the base material could be called high or even ultra-high performance concrete. However, for the purpose of brevity, we denominate it as *concrete* herein.

The concrete PA was reinforced with 40 kg/m${}^{3}$ straight steel fibers of 13 mm in length, 0.2 mm in diameter (Dramix OL 13/0.20, 65 in aspect ratio), the slump flow reached 70 cm × 70 cm in cross–direction diameters. In contrast, the concrete PB and PC were additionally reinforced with long hooked-end steel fibers of 30 mm in length, 0.38 in diameter (RC 80/30 BP, 80 in aspect ratio) of 20 and 60 kg/m${}^{3}$, respectively. The corresponding slump flow tests for PB and PC reached 66 cm × 67 cm and 57 cm × 57 cm. Characterization tests were carried out using cylindrical specimens of 150 mm × 300 mm (diameter × height) to measure the compressive strength at the age of seven months. The results are given in Table 1. Additionally listed in the table are the measured elastic modulus (*E*), Poisson’s ratio (ν) and the material density (ρ). Furthermore, the flexural strength (or the first-crack strength, the modulus of rupture), $f_{R}$, can be estimated through a three-point bending test (to be explained in the next subsection) as follows,

(1) $$f_{R}=\frac{3P_{ini}\;S}{2B{\left(D-a\right)}^{2}}$$

where $P_{ini}$ is the load upon crack initiation from the notch, *S*, *D*, *B* and *a* are the beam span, depth, width and notch height, respectively. In Table 1, values of $f_{R}$ measured at the loading-displacement rate of 2.2 μm/s (quasi-static) are reported. Note that even though the compressive strength ($f_{c}$) and the elastic modulus (*E*) remain practically the same for the three types of concrete, significant improvement of the flexural strength in PC compared to PA and PB is observed.

Terminal crack velocities are of importance in some applications [17]. If PA, PB and PC are considered homogeneous and isotropic, the longitudinal wave velocity, $C_{L}$, can be calculated as $\sqrt{E/\rho }$, the terminal crack velocity, $v_{tc}$, estimated as $0.38C_{L}$ according to the state-of-the art study by Quinn [17]. Both values are listed in Table 1 to facilitate further discussion in Section 3.

### 2.2. Three-Point Bending Tests

**Figure 1:**
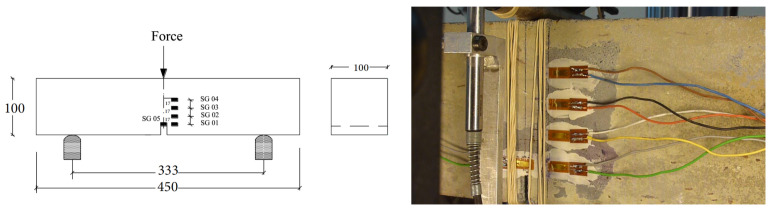
Beam geometry and the distribution of the glued strain gages.

Three-point bending tests were carried out on notched beams of all three types of concrete according to the RILEM standard [20]. Thirteen beams were prepared for each material type. Each beam was denominated as PXn, where PX represents the material type PA, PB or PC, whereas n is the specimen numbering. Specifically, the beams labelled as PX2, PX3, PX4 were assigned for loading at 2.2 μm/s, PX7, PX8, PX9 for 0.22 mm/s, PX12, PX13, PX14 for impact tests at 1.77 m/s and PX17, PX18, PX19 for tests at 2.66 m/s, respectively. In addition, the PX15 beams were also tested at 2.66 m/s with the difference that a high-speed camera was setup for posterior DIC analysis. All beams were 100 mm × 100 mm in cross section, 450 mm in length (to be tested with a span of 333 mm), the central notch was 17 mm in height, see Figure 1.

After surface polishing, four strain gages (type LY 116/120A) of 6 mm in length, 2.8 mm in width, were glued to the specimen 13 mm away from the central line. A fifth gage was glued right in front of the notch to capture the strain rate at crack initiation. These gages were named as SG1, SG2, SG3, SG4 and SG5 as shown in Figure 1. The HBM MGCplus system (strain amplifier and integrated oscilloscope) was used for data acquisition from the strain gages. The sample rate was set at 100 Hz and 2.4 kHz for the tests using a servo-hydraulic machine at 2.2 μm/s and 22 mm/s, respectively.

**Figure 2:**
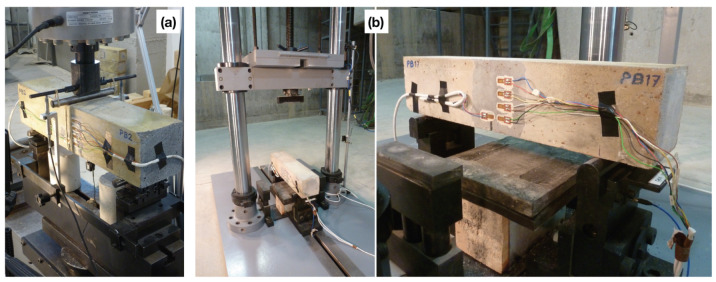
(**a**) A beam prepared for testing using the servo-hydraulic testing machine; and (**b**) experimental set-up using the drop-weight impact device, the strain gages were connected to the individual channels of the data acquisition system.

A drop-weight impact device [21], see Figure 2, was utilized to drop a hammer of 120.6 kg from a height of 160 mm and 360 mm to achieve an impact velocity of 1.77 m/s and 2.66 m/s, respectively. A piezoelectric force sensor recorded the impact force; two force sensors between the support and the specimen registered the reaction forces. A DEWETRON-30-8 strain amplifier and two TDS3014B oscilloscopes were used to acquire data from the strain gages with a sample rate set at 250 kHz, which resulted a time interval of 4 μs between two adjacent gage readings.

#### 2.2.1. Measuring the Load and Strain Rate upon Crack Initiation

As mentioned above, the strain gage SG1 is glued to the beam surface at the same height as that of the notch tip but with a horizontal distance of 13 mm. Therefore, the shortest time that a stress wave needs to travel from the notch tip to SG1 is less than 3 μs whereas the time interval between adjacent strain readings is 4 μs. Consequently, we can neglect the time delay of the gage readings between SG1 and SG5. The strain peak observed in SG1 can be subsequently used to determine the load upon the crack initiation from the notch tip.

**Figure 3:**
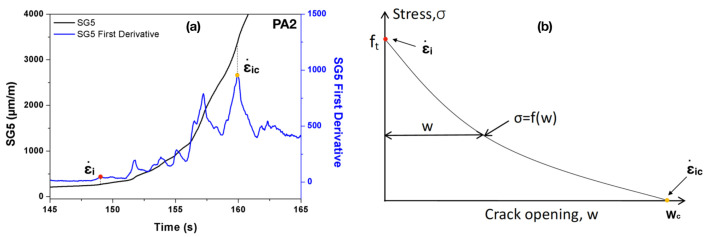
Determining the strain rate upon crack initiation using (**a**) the strain history data recorded in SG5 (shown for PA2 loaded at 22 μm/s); and (**b**) a generic cohesive law.

From the recordings of the strain gage SG5, we can take the first derivative with respect to time and obtain the corresponding strain rate evolutions, see Figure 3a. Based on the cohesive theories of fracture, we expect an abrupt change in the strain rate curve for each abrupt slope change of the softening curve given in Figure 3b. The maximum strain variation should occur at the formation of a stress-free crack tip, i.e., when the critical crack opening displacement, $w_{c}$, is obtained. We denominate the corresponding strain rate as ${\dot{\varepsilon }}_{ic}=\dot{\varepsilon }\left(w_{c}\right)$. In the same way, the first peak in the $\dot{\varepsilon }$–time curve can be identified as the emergence of the cohesive crack tip, when the crack opening displacement is still null, i.e., ${\dot{\varepsilon }}_{i}=\dot{\varepsilon }\left(0\right)$.

Since the matrix tensile strength was not measured, we approximate its value as one tenth of the compressive strength of PA given in Table 1. Consequently, the maximum tensile strain can be taken as roughly 250 με. This value is taken as an additional reference in locating the strain rate upon the initiation of a cohesive crack tip.

#### 2.2.2. Quantification of the Energy Absorption Capacity

**Figure 4:**
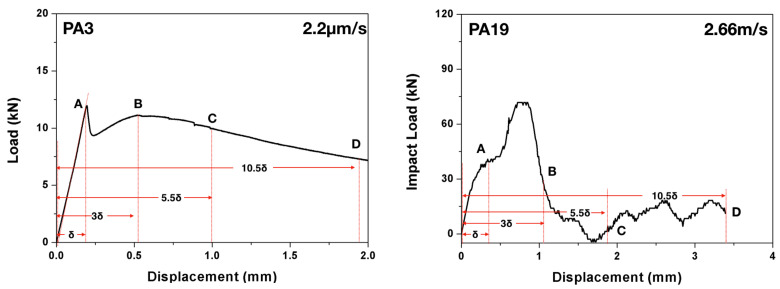
Measuring the toughness indices using the load-deflection curve (shown for PA3 loaded at 2.2 μ/s and PA19 impacted at 2.66 m/s).

According to the ASTM standard C1018-97 [22], three-point bending tests can be used to evaluate the flexural performance of toughness parameters derived from fiber-reinforced concrete in terms of areas under the obtained load-deflection curve. The toughness determined in this manner is an indication of the energy absorption capability of the particular test specimen, whereas the toughness indices (the numbers obtained dividing the area up to a specified deflection criterion by the area up to the first crack in the matrix) are independent of geometrical specimen and testing variables (such as the span length). Specifically, the toughness indices, $I_{5}$, $I_{10}$, $I_{20}$, are obtained by dividing the area up to a deflection of respective 3.0, 5.5 and 10.5 times the first-crack deflection (δ) by the area up to the first crack, see Figure 4 in the case of PA3 and PA19 for details. The residual strength factors, which represent the average post-peak load retained over a specific deflection interval as a percentage of the load at first crack, can be subsequently calculated. In particular, $R_{5,10}$ and $R_{10,20}$ are defined as $20\;(I_{10}-I_{5})$ and $10\;(I_{20}-I_{10})$, respectively.

It needs to be pointed out that, the concept of toughness indices and residual strength factors are defined in the ASTM standard [22] solely for static flexural tests. In the current work, we extend it to dynamic loading conditions by applying the above procedure to the impact load–deflection curves. It bears emphasis that, under impact loading, since the first-crack initiation was detected through the strain gage SG1, it does not necessarily coincide with the change of slope of the impact load-displacement curve, for example in the case of PA19 shown in Figure 4.

#### 2.2.3. Measuring the Crack Propagation Velocity Using the Strain Gage Readings

**Figure 5:**
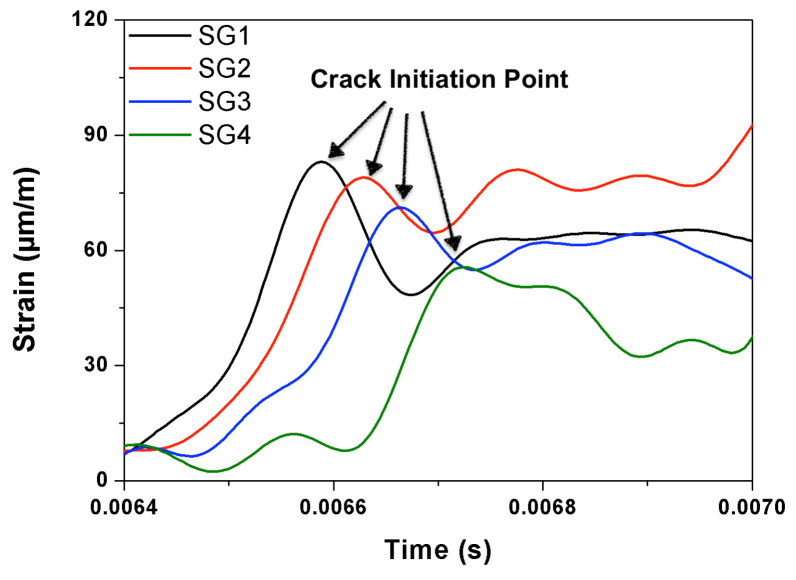
Typical strain-history curves of a PA specimen recorded in SG1–SG4 (loading rate: 2.66 m/s).

During the process of crack initiation and propagation, the stress (and strain) around the crack tip is relaxed, such a relaxation process can be recorded by a strain gage as the appearance of a strain peak in the strain versus time curve. If multiple gages were closely placed on a path parallel to that of the expected crack path, the average propagation velocity between two adjacent gages can be calculated accordingly. In the current work, four strain gages distanced at 17 mm from each other as shown in Figure 1 and Figure 2, were glued at the central part of the notched beam. Therefore, four values of the crack speed can be obtained. As an example, the strain histories of the strain gages SG1–SG4 for concrete PA impacted at 2.66 m/s are given in Figure 5. Note that the crack initiated from the notch tip at the time when the strain recorded in SG1 reached its maximum, whereas the strain peaks recorded in SG2, SG3 and SG4 indicated the moments when this same crack passed through the positions marked by these strain gages glued at the same heights.

#### 2.2.4. Crack Velocity Measurement Using DIC

Strain can also be measured using digital image correlation (DIC). One or two cameras are normally used in conjunction with a DIC software to track features on the specimen surface to detect small motions. The technique of DIC was developed in the 1980s [23,24] as a combination of digital image processing technology and optical mechanics. It uses the DIC software to analyze the data of the random speckle distribution on the surface recorded in a series of digital images. By comparing to a reference image, the gray values of the current digital image can be used to accurately measure the deformation and displacement [25].

**Figure 6:**
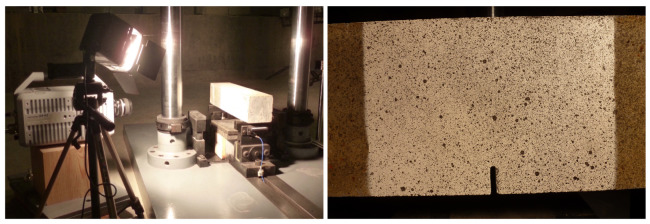
The experimental setup with the high-speed camera (**left**), and the speckle pattern obtained from black/white paint using air spray (**right**).

In the current study, the high-speed camera PHOTRON FASTCAM SA-Z 2100K-M-8Gb was used to capture images of crack growth with a sample rate of 27,000 frames per second. Sub-images of 17 × 17 pixels were regularly spaced into a grid in the reference image (640 × 696 pixels in size). A speckle pattern of black and white paint was air sprayed onto the central part of the specimen surface, where the mode I crack was expected to grow, see Figure 6.

For clarity, we summarize the beams to be tested under three-point bending configuration under four loading rates in Table 2, the ones glued with strain gages will be used to measure parameters related with external loading, $P_{max}$ and $P_{ini}$ (thus the first crack strength, $f_{R}$), the strain rates upon crack intitiation, ${\dot{\varepsilon }}_{i}$ and ${\dot{\varepsilon }}_{ic}$, the energy absorption, $I_{5},I_{10},\;I_{20},\;R_{5,10}$ and $R_{10,20}$, as well as the crack speed, $V_{SG}$. The beams designated for testing with a high-speed camera, are mainly used for complementary measurement of the crack speed with DIC.

## 3. Results and Discussion

In this Section, the crack initiation load detected from SG1 together with the peak load, the apparent fracture energy and the strain rate at crack initiation are first reported. Next, the failure patterns of beams made from all three types of concrete PA, PB and PC, loaded at four loading rates are presented. The measured crack velocities using strain gages and preliminary measurement using the technique of DIC are subsequently given. Finally, results obtained from the numerical simulations are illustrated.

### 3.1. Load and Strain Rates upon Crack Initiation

**Figure 7:**
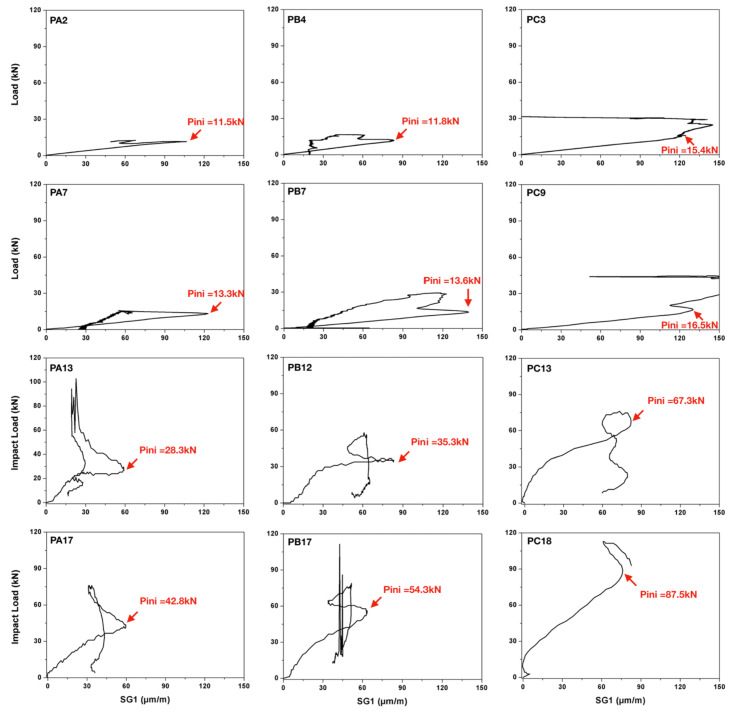
Typical load versus-strain curves recorded in SG1 for beams made from concrete PA (**left**), PB (**central**) and PC (**right**) loaded at 22 μm/s (the 1st row), 22 mm/s (the 2nd row), 1.77 m/s (the 3rd row) and 2.66 m/s (the 4th row), respectively.

In Figure 7, typical load-strain curves (recorded in SG1) for concrete PA, PB and PC under four loading rates are plotted. As mentioned before, upon crack initiation, the stress (strain) concentration at the crack tip is relaxed, consequently, a local maximum of the strain can be recorded in the gage, the corresponding load is the so-called crack initiation load $P_{ini}$. The measured crack initiation load $P_{ini}$ together with the peak load $P_{max}$, the corresponding mean values and standard deviations are listed in Table 3. Note that in Figure 7, clear strain peaks can be identified for PA and PB. However, due to the high fiber content, a local strain maximum is hardly perceived for PC. This difficulty makes the identification of crack initiation time less reliable, thus those measurements based on the localization of the local strain maximums, such as $P_{ini}$ and the crack velocity, will inevitably involve larger dispersions.

**Figure 8:**
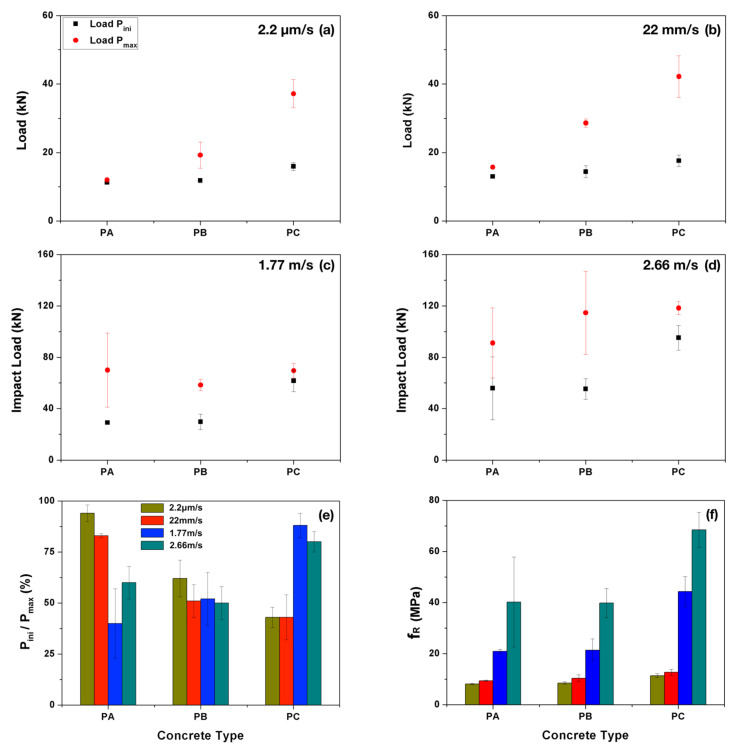
Peak load and initial load (with respective standard deviations) versus concrete type for the loading rate of (**a**) 2.2 μm/s; (**b**) 22 mm/s; (**c**) 1.77 m/s and (**d**) 2.66 m/s, respectively; (**e**) the ratio between both loads; and (**f**) the first-crack strength, $f_{R}$.

In Figure 8, both the maximum impact load and the crack initiation load are plotted for each concrete type at each loading rate. From the figure, it is observed that both loads increase with the loading rate. When the loading rate is low, such as 2.2 μm/s and 22 mm/s, these two load values further augmented with the fiber content. Under impact loading, however, such effect is not clearly identified. The ratios between the initial load and the peak load at different loading rates are presented. At low loading rates, this ratio is observed to decrease significantly with the fiber content. Under impact loading, however, the ratio is relatively low for both PA and PB, whereas for PC, no clear trends can be detected. The more extended error bands for PC, see Figure 8e, confirm the larger dispersions in PC than those of PA and PB.

Once the crack initiation loads are obtained, the corresponding first crack-strength, $f_{R}$, can be calculated using Equation (1). The values of $f_{R}$ for each concrete type at four different loading rates are reported in Table 4 and plotted in Figure 8e. Note that the first-frack strength is significantly improved under impact loading for all three types of concrete, whereas at the same loading rate, PC gives the highest first-crack strength.

**Figure 9:**
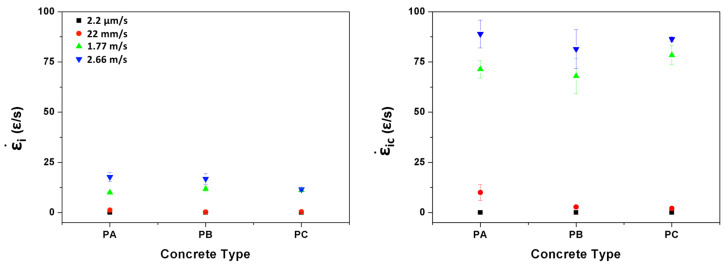
The strain rates ${\dot{\varepsilon }}_{i}$ and ${\dot{\varepsilon }}_{ic}$ upon crack initiation at the notch tip.

Additionally given in Table 3 are the strain rates upon the initiation of a cohesive crack, ${\dot{\varepsilon }}_{i}$, and upon formation of a stress-free crack, ${\dot{\varepsilon }}_{ic}$. These results are also represented in Figure 9. Note that both strain rates slightly decreased with the increase of fiber content at low loading rates, whereas no significant variations can be perceived under high loading rates. It bears emphasis that the values of ${\dot{\varepsilon }}_{i}$ are similar to the strain rate obtained in the work of Zhang et al. [6]. However, the strain rates upon the formation of a stress-free crack, ${\dot{\varepsilon }}_{ic}$, are not reported in [6]. In addition, both strain rates measured herein cover seven orders of magnitude, specifically ${\dot{\varepsilon }}_{i}$, ranged from 10${}^{-5}$ to 10 s${}^{-1}$ for ${\dot{\varepsilon }}_{i}$, whereas ${\dot{\varepsilon }}_{ic}$ varied from 10${}^{-4}$ to 10${}^{2}$ s${}^{-1}$.

### 3.2. The Failure Patterns, Measured Toughness Indices and Residual Strength Factors

**Figure 10:**
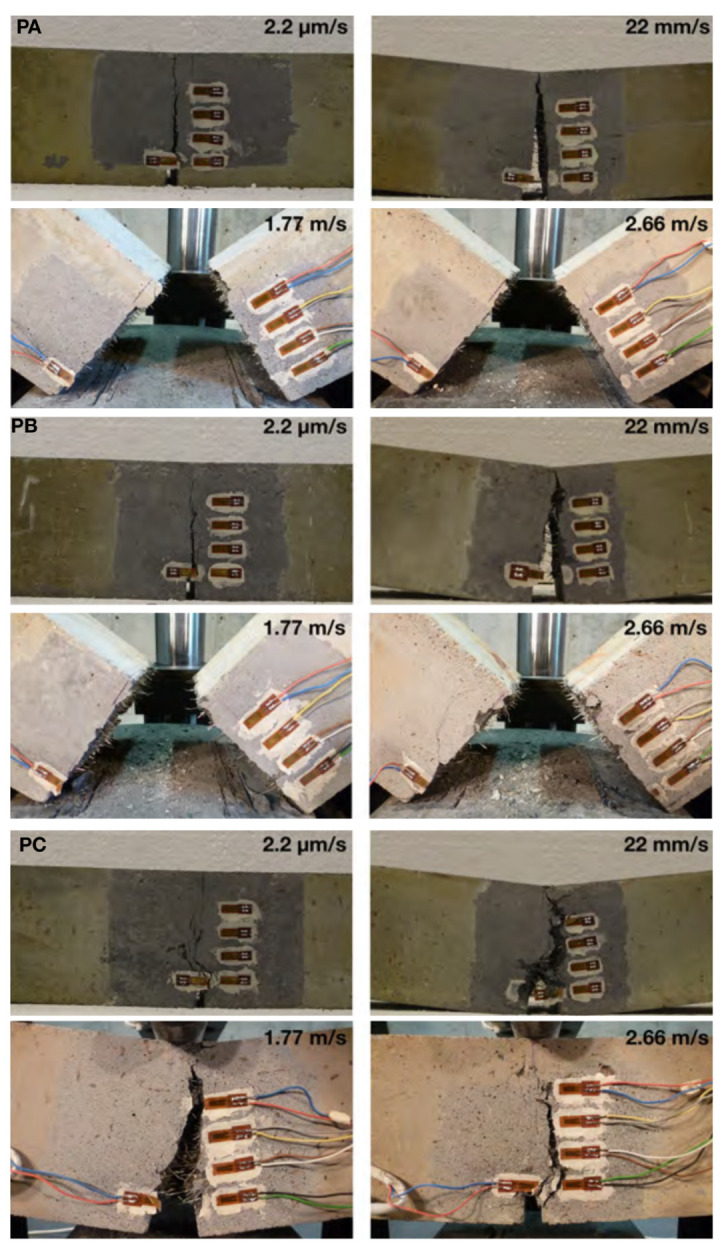
Typical failure modes of the PA, PB and PC beams loaded under four different loading rates.

The images of the broken specimens made from the concrete PA, PB and PC at four different loading rates are given in Figure 10. For PA and PB beams, one main crack started at the notch tip and propagated towards the loading point in spite of the fiber content and the loading rate. The difference lies in the fact that, at lower loading rates, this crack did not go through the entire beam whereas at impact loading, the beam broke into two parts. Note that with more fibers, the PB beams present more mist or hackle cracks around the main crack. In addition, the steel fibers are pulled out at the crack surface.

**Figure 11:**
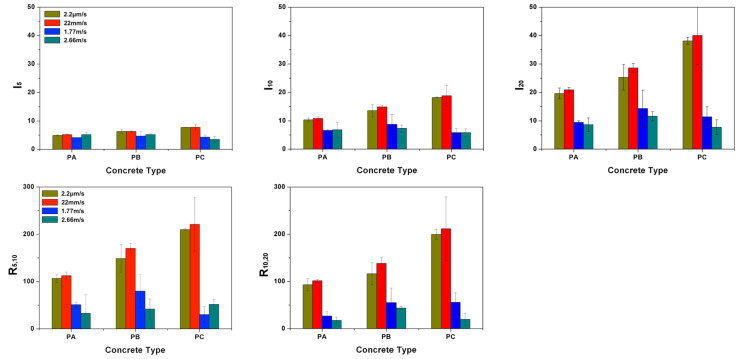
The toughness indices, $I_{5}$, $I_{10}$, $I_{20}$, and the residual strength factors, $R_{5,10}$ and $R_{20,10}$.

In the case of concrete PC, the beams were not broken even under impact loading due to the fact that the energy supplied by dropping the 120-kg hammer from a height of either 160 mm or 360 mm was not sufficient to break the PC beams. The main crack was more tortuous with numerous micro-cracks formed. The calculated toughness indices, $I_{5}$, $I_{10}$ and $I_{20}$, the residual strength factors, $R_{5,10}$ and $R_{10,20}$, calculated for concrete PA, PB and PC at four different loading rates are given in Table 5. These results are also plotted in Figure 11 for a better visualization. From Figure 11, it can be observed that, at low loading rates, the energy absorption capability is greatly enhanced with the fiber addition, it is particularly so for PC, which has the highest fiber content. For instance, the values of $I_{20}$ for PC beams reached 40 when loaded at 22 mm/s, this indicates, at the deflection of 10.5δ, the beam had absorbed 40 times the energy absorbed at the deflection of δ, when the first crack was formed. Similar toughness indices were reported by Ríos et al. [26] for ultra-high performance concrete with polypropylene fibers tested at room temperature. By contrast, the values of $I_{20}$ for PA and PB reached 20 and 30, respectively. Having in mind that a value of 20 for $I_{20}$ represents a perfectly plastic behavior in average, all three material obtained a strain-hardening tendency accounting from the first-crack formation. This is further confirmed by the residual strength factors, $R_{5,10}$ and $R_{10,20}$, shown in the same figure, in which values greater, equal or less than 100% represent a strain hardening, a perfectly plastic or softening behavior, respectively.

### 3.3. The Measured Crack Propagation Velocity Using the Strain Gages

**Figure 12:**
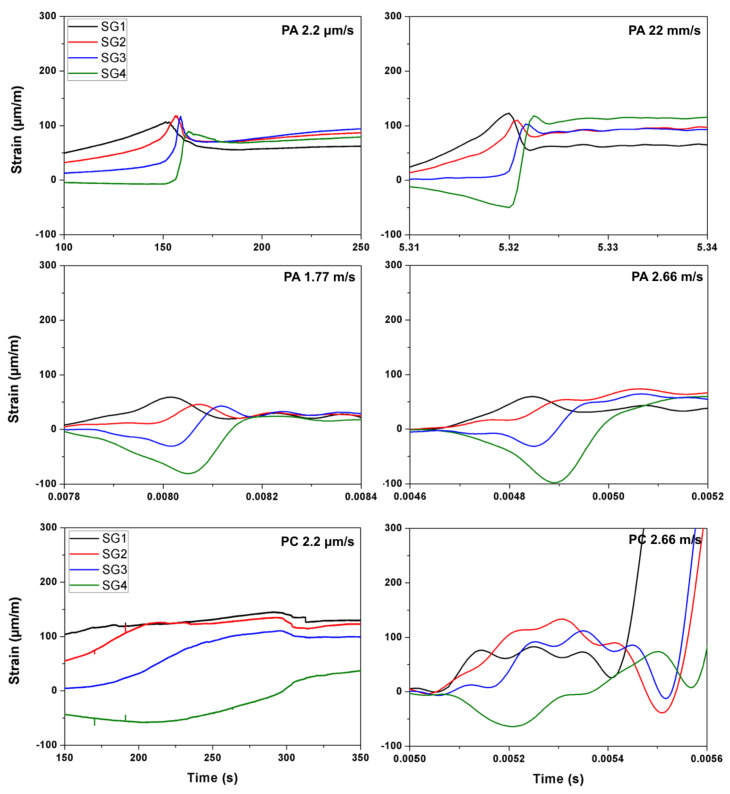
Typical strain histories for concrete PA (at four loading rates) and PC (at the lowest and highest loading rates only).

**Figure 13:**
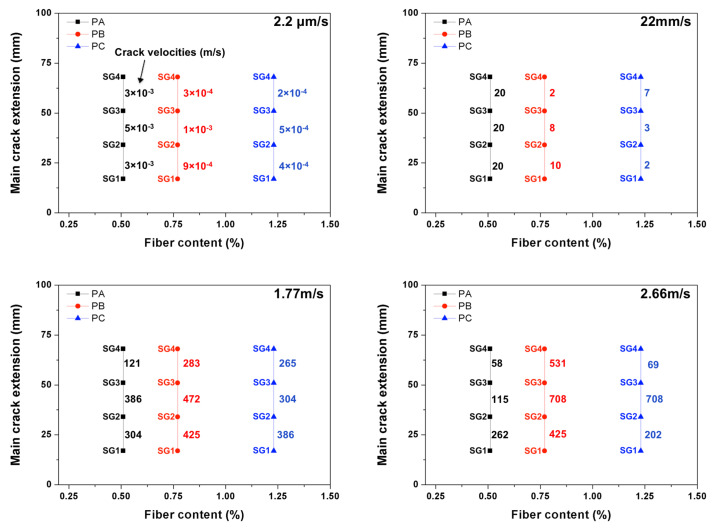
Typical crack velocities marked (in m/s) along the main crack path for concrete PA, PB and PC loaded at four loading rates: 2.2 μm/s, 22 m/s, 1.77 m/s and 2.66 m/s, respectively.

For the convenience of further discussion, the typical strain histories recorded in the gages SG1, SG2, SG3 and SG4 under selected loading rates are presented in Figure 12 for PA and PC. The measured mean crack speed values between two adjacent strain gages are listed in Table 6 and Table 7, the maximum speed obtained are marked out as bold numbers. In addition, due to the fact that the strain gages glued on PB3, PB9 and PA12 failed to record any data, no velocity information was obtained for these three beams. Note that, at the quasi-static loading rate, the main crack speed was in the order of mm/s, the maximum value (15.6 mm/s) appeared in the PA specimens. With the increase of fiber content, the crack propagation slowed down by almost one order of magnitude. When loaded at 22 mm/s, the crack speed reached a maximum of 21 m/s for PA, 14 m/s for PB and 10 m/s for PC, respectively. Under impact loading, the maximum values of 1063 m/s for PA, 708 m/s for PB and 1417 m/s for PC were obtained. In addition, the typical crack velocities marked along the main crack path for concrete PA, PB and PC beams loaded at four different rates are plotted in Figure 13.

### 3.4. Measuring the Crack Propagation Velocity Using DIC

For the purpose of contrasting the velocity data obtained with strain gage readings, three beams (PA15, PB15 and PC15) were impacted at 2.66 m/s, their failing process recorded with a high-speed camera. In order to calculate the crack propagation velocity, the crack tip needs to be localized for the time when the image was taken. Assuming a generic cohesive law, see Figure 3b, the cohesive crack tip is defined as the point when the first principal stress reached the tensile strength of the matrix. In practice, in order to reduce the influence of the background noise often present in the DIC images, a small value of the crack opening displacement $w_{0}$, in our case, 2.5 μm (comparable to the 2 μm used by Chen and Su [27] for concrete specimens), is used to identify the cohesive crack tip. Since the material is considered as elastic until fracture, the crack tip can also be located at the point when the maximum tensile strain is reached as suggested by Wu et al. [28].

**Figure 14:**
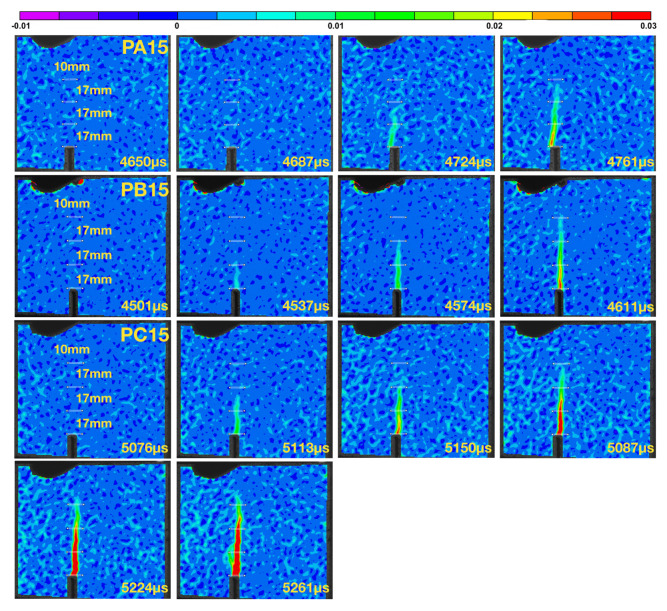
The crack propagation captured using a high-speed camera and processed using a DIC software as strain contours for the specimens PA15, PB15 and PC15 impacted at 2.66 m/s.

The crack opening displacements were collected at three positions which are considered as virtual extensometers dedicated to determine the crack propagation velocity. The strain distribution shown as snap shots of the DIC images are presented in Figure 14. The obtained crack speeds given in Table 8 compare quite well with those measured using the strain gage readings.

### 3.5. Numerical Simulations Based on the Cohesive Theories of Fracture

In this section, based on the cohesive theories of fracture [18,29], a single crack initiated from the crack tip is modeled. The SFRSCC material, in spite of its fiber content, is considered homogeneous and linear elastic until fracture. In other words, instead of an explicit representation [30], the steel fibers are implicitly taken into account to save computational efforts. After fracture, a linear decreasing cohesive law governs the opening behavior of the cohesive crack. The flexural strength given in Table 1, is taken as the material’s tensile strength, whereas the apparent fracture energy measured for the deflection up to 3 mm reported by Zhang et al. [19] is used as the specific fracture energy at each loading rate, in other words, the strain-rate effect is implicitly included in the material parameters. The experimental impact–time history is fed to the numerical model as the load input. The zones without expected fracture are represented as four-node solid elements, whereas the crack is simulated as pairs of contact elements with cohesive nature in traction. The model is implemented in the commercial software Ansys, similar to the work of Poveda et al. [31]. The experimental impact loads are used as input data for beams impacted at 1.77 and 2.66 m/s.

**Figure 15:**
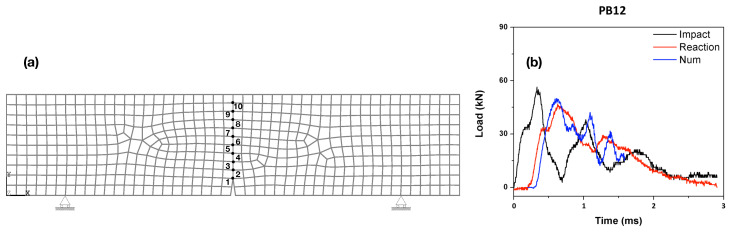
(**a**) Distribution of mesh in the specimen and ligament nodes; (**b**) evolution of the numerical and experimental reaction forces contrasted with the impact forces at loading rate of 1.77 m/s (PB12).

A typical mesh used in the simulations is illustrated in Figure 15a, where the points at the central segment are marked out for the crack speed. It needs to be pointed out, the mesh-dependency issue has been thoroughly studied previously [31,32,33,34,35,36,37,38], and it has been concluded that with a mesh size commensurate with that of the maximum aggregate size (or the representative length at the mesoscale), the numerical model based on the cohesive theories of fracture is capable of produce mesh-independent results. Since all three types of material PA, PB and PC are considered homogeneous, the short steel fibers were 13 mm in length, the mesh size of 7.5 mm adopted in Figure 15a can indeed produce mesh-independent results. For the purpose of validation, the numerically obtained reaction forces are plotted together with the experimental ones in Figure 15b for the beam PB12 loaded at 1.77 m/s. Simulations were performed for all the beams with their corresponding impact loads as input, the obtained crack velocities between adjacent points are listed in Table 9. Note that, speeds up to 589, 401 and 1016 m/s are obtained for PA, PB and PC beams, respectively.

### 3.6. Comparison of the Obtained Crack Velocities

**Figure 16:**
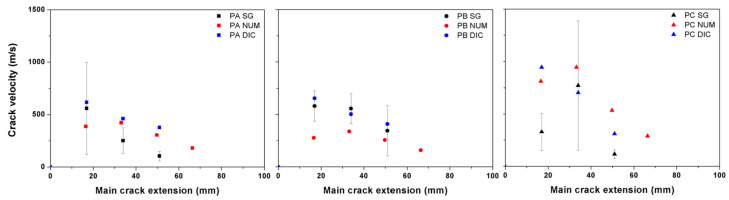
Comparison of the obtained crack velocities using strain gages (SG), digital image Correlation (DIC) and numerical simulations (NUM) for PA, PB and PC beams impacted at 2.66 m/s.

In Figure 16, the obtained crack velocities for PA, PB and PC beams impacted at 2.66 m/s from the strain gages, the technique of DIC and numerical simulations are compared. As a general trend, as the crack advanced further, its speed decreased except in the case of PC. This can be explained by the fact that, the rather high fiber content in PC may inevitably modify its internal structure. Consequently, a more uniform fiber distribution is more difficult to reach, the material PC is less homogeneous in comparison with PA and PB. In other words, the more fiber content does significantly enhance the material’s energy absorption capacity, however, it does not necessarily slow down the main crack propagation.

As mentioned before, the crack velocities are considered to be limited by the terminal crack velocity, $v_{ct}$. For a homogeneous and isotropic material, it can be estimated as up to 38% of the material’s longitudinal wave velocity. It is noteworthy that, the maximum speed measured in PC, 1417 m/s, is fairly close to the terminal crack velocity 1600–1700 m/s given in Table 1. It is rather close to the maximum crack speed of 1454 m/s reported by Pyo et al. [14] for a steel fiber-reinforced ultra high-performance concrete.

## 4. Conclusions

Three-point bending tests have been carried out on three types of steel-fiber reinforced self-compacting concrete under four different loading rates. Two types of steel fibers were added. One is 13 mm in length, 0.2 mm in diameter, the other is 30 mm in length, 0.38 mm in diameter. The effect of fiber content (0.77%, 0.51% and 1.23%) and loading rate (2.2 μm/s, 22 mm/s, 1.77 m/s and 2.66 m/s) on the mode-I crack advancing velocity is explored using the technique of strain gages and complemented with the DIC measurement and numerical simulations.

At low loading rates, fiber addition is observed to slow down the crack propagation. Under impact loading, such an effect is not clearly perceived. By contrast, the highest crack speed of 1417m/s was measured in a PC beam impacted at 2.66 m/s. The second fastest speed of 1063 m/s was obtained in a PA beam. It is noteworthy that, they are fairly close to the terminal crack velocity 1600–1700 m/s if the three types of concrete were considered homogeneous and isotropic. It will be of great interest to verify if such a high crack speed is also attainable in a mixed-mode framework. This topic will be reported in a future contribution.

The concept of toughness indices and residual strength factors are employed to quantify the energy absorption capacity for fiber reinforced concrete under dynamic loading. It is shown that this capacity is significantly enhanced by fiber addition. This is manifest with increased micro cracks and more tortuous crack path in the PC beams.

Both strain rates, one is upon the initiation of a cohesive crack (when the opening displacement is null), ${\dot{\varepsilon }}_{i}$, the other is at the formation of a stress-free crack (when the critical opening displacement is obtained), ${\dot{\varepsilon }}_{ic}$, are measured using the strain gage glued at the notch tip. Both covered seven orders of magnitude, the former ranged from 20 με/s for the quasi-static loading to 20 s${}^{-1}$ under impact loading, whereas the latter varied from 500 με/s to 100 s${}^{-1}$.

## Figures and Tables

**Table 1:** Properties of concretes at the age of seven months (values in parentheses are the standard deviations), the longitudinal wave velocity, $C_{L}$, and the estimated terminal crack velocity, $v_{ct}$.

	$f_{c}$ (MPa)	$f_{R}$ (MPa)	*E* (GPa)	ν -	ρ (kg/m${}^{3}$)	$C_{L}$ (m/s)	$v_{\mathrm{ct}}$ (m/s)
PA	112 (1)	8.1 (0.2)	46.4 (3.0)	0.18 (0.01)	2362 (3)	4432	1684
PB	112 (6)	8.5 (0.5)	45.2 (2.0)	0.17 (0.01)	2376 (32)	4362	1658
PC	114 (3)	11.4 (0.8)	45.9 (3.0)	0.17 (0.01)	2408 (11)	4366	1659

**Table 2:** Three-point bending tests loading rate with tested beam ID, the corresponding fiber content. The beams with glued strain gages are designed to measure $P_{max}$, $P_{ini}$, $f_{R}$, ${\dot{\varepsilon }}_{i},{\dot{\varepsilon }}_{ic}$, $I_{5},\;I_{10},\;I_{20},\;R_{5,10},\;R_{10,20}$ and $V_{SG}$, whereas the three beams PA15, PB15 and PC15 were assigned to DIC analysis to measure the crack speed.

Loading Rate (mm/s)	Beam ID	Fiber Content	Gages or DIC
0.0022	PA2, PA3, PA4	0.51%	
22	PA7, PA8, PA9	0.51%	Strain gages
1770	PA12, PA13, PA14	0.51%	
2660	PA17, PA18, PA19	0.51%	
2660	PA15	0.51%	DIC
0.0022	PB2, PB3, PB4	0.77%	
22	PB7, PB8, PB9	0.77%	Strain gages
1770	PB12, PB13, PB14	0.77%	
2660	PB17, PB18, PB19	0.77%	
2660	PB15	0.77%	DIC
0.0022	PC2, PC3, PC4	1.23%	
22	PC7, PC8, PC9	1.23%	Strain gages
1770	PC12, PC13, PC14	1.23%	
2660	PC17, PC18, PC19	1.23%	
2660	PC15	1.23%	DIC

**Table 3:** Measured impact load $P_{max}$, $P_{ini}$, the ratios between both, the strain rate upon the initiation of a cohesive crack ${\dot{\varepsilon }}_{i}$, and the strain rate upon the formation of a stress-free crack ${\dot{\varepsilon }}_{ic}$, the corresponding mean values with standard deviations shown in the parentheses are given in the fourth row.

Beam No.	Loading Rate (mm/s)	$P_{\mathrm{ini}}$ (kN)	$P_{\max}$ (kN)	$P_{\mathrm{ini}}/P_{\max}$ (%)	${\dot{\varepsilon }}_{i}$ (με/s)	${\dot{\varepsilon }}_{\mathrm{ic}}$ (με/s)
PA2		11.5	12.6	91	48	950
PA3	0.0022	11.1	12.0	92	300	3300
PA4		11.2	11.4	99	84	980
		11.3 (0.2)	12.0 (0.6)	94 (4)	144 (136)	1743 (1348)
PB2		12.5	23.8	53	26	740
PB3	0.0022	11.2	17.6	64	38	480
PB4		11.8	16.7	71	51	550
		11.9 (0.7)	19.4 (3.8)	62 (9)	38 (13)	590 (135)
PC2		17.2	39.6	44	45	530
PC3	0.0022	15.4	32.4	47	49	780
PC4		15.1	39.5	38	46	480
		15.9 (1)	37.2 (4.1)	43 (5)	47 (2)	597 (161)
					(ε/s)	(ε/s)
PA7		13.3	16.0	83	1.1	8.5
PA8	22	13.3	16.0	83	0.6	7.6
PA9		12.5	15.2	82	1.9	15.0
		13.0 (0.5)	15.8 (0.5)	83 (1)	1.2 (0.7)	10.0 (4.0)
PB7		13.6	29.4	46	0.2	2.9
PB8	22	13.2	29.1	45	0.3	3.1
PB9		16.4	27.2	60	0.3	2.4
		14.4 (2)	28.6 (1.2)	51 (8)	0.3 (0.1)	2.8 (0.4)
PC7		19.5	35.2	56	0.2	1.4
PC8	22	16.8	46.5	36	0.6	2.9
PC9		16.5	44.8	37	0.3	1.6
		17.6 (1.7)	42.2 (6.1)	43 (11)	0.4 (0.2)	2 (0.8)
PA12		-	49.3	-	10.0	73.0
PA13	1770	28.3	102.8	28	10.1	74.2
PA14		29.8	57.8	52	9.8	66.4
		29.1 (1.1)	70.0 (28.8)	40 (17)	10 (0.2)	71.3 (4.3)
PB12		35.3	57.0	62	13.1	65.0
PB13	1770	23.3	63.4	37	10.3	77.8
PB14		30.6	54.9	56	11.9	61.0
		29.7 (6.0)	58.5 (4.4)	52 (13)	11.8 (1.4)	67.9 (8.8)
PC12		65.5	70.4	93	10.9	83.6
PC13	1770	67.2	74.7	90	11.9	77.7
PC14		52.1	63.4	82	10.4	74.0
		61.6 (8.3)	69.5 (6.0)	88 (6)	11.1 (0.8)	78.4 (4.8)
PA17		42.8	77.5	55	16.8	85.2
PA18	2660	84.1	122.5	69	16.1	96.8
PA19		40.8	73.2	56	20.3	84.6
		55.9 (24.4)	91.1 (27)	60 (8)	17.7 (2.3)	88.9 (6.9)
PB17		54.3	112.7	48	19.7	79.1
PB18	2660	63.7	147.9	43	16.1	92.0
PB19		47.8	83.1	53	14.5	73.0
		55.3 (8)	114.6 (32.4)	50 (8)	16.8 (2.7)	81.4 (9.7)
PC17		105.9	122.5	86	12.4	86.4
PC18	2660	87.5	112.7	78	10.9	85.2
PC19		91.8	119.7	77	11.3	87.4
		95.1 (9.6)	118.3 (5.1)	80 (5)	11.5 (0.8)	86.3 (1.1)

**Table 4:** The measured first-crack strength, $f_{R}$, in MPa, at four different loading-displacement rates for concrete PA, PB and PC.

Concrete Type	2.2 (μm/s)	22 (mm/s)	1.77 (m/s)	2.66 (m/s)
PA	8.1 (0.2)	9.4 (0.3)	20.9 (0.8)	40.2 (17.6)
PB	8.5 (0.5)	10.4 (1.3)	21.4 (4.4)	39.8 (5.7)
PC	11.4 (0.8)	12.7 (1.2)	44.3 (5.9)	68.4 (6.9)

**Table 5:** The measured toughness indices and residual strength factors with their corresponding standard deviations in the parentheses for the three types of concrete at four different loading rates.

Concrete	Loading (mm/s)	$I_{5}$	$I_{10}$	$I_{20}$	$R_{5,10}$	$R_{10,20}$
PA		4.9 (0.2)	10.3 (0.6)	19.6 (1.9)	107 (9)	93 (13)
PB	0.0022	6.3 (0.7)	13.6 (2.2)	25.3 (4.5)	149 (30)	116 (24)
PC		7.7 (0.1)	18.2 (0.2)	38.1 (1.2)	210 (3)	200 (10)
PA		5.2 (0.2)	10.8 (0.6)	20.9 (0.8)	113 (7)	101 (3)
PB	22	6.3 (0.2)	14.9 (0.6)	28.6 (1.5)	170 (11)	138 (12)
PC		7.8 (1.0)	18.8 (3.7)	40 (10.3)	221 (57)	212 (68)
PA		4.1 (0.0)	6.6 (0.3)	9.4 (0.6)	51 (5)	27 (9)
PB	1770	4.7 (1.7)	8.7 (3.5)	14.3 (6.5)	80 (35)	55 (31)
PC		4.3 (0.7)	5.8 (1.5)	11.4 (3.5)	30 (17)	56 (20)
PA		5.2 (0.8)	6.8 (2.7)	8.6 (2.4)	33 (39)	18 (7)
PB	2660	5.2 (0.3)	7.3 (1.2)	11.6 (1.6)	42 (22)	44 (4)
PC		3.5 (1.1)	5.8 (1.4)	7.7 (2.6)	52 (10)	20 (13)

**Table 6:** Measured crack speed for the quasi-static loading rate of 2.2 μm/s (specimens PX2, PX3 and PX4) and 22 mm/s (specimens PX7, PX8 and PX9), the corresponding mean and standard deviations are given in the fourth row of each concrete type.

Beam	$V_{SG12}$ (mm/s)	$V_{SG23}$ (mm/s)	$V_{SG34}$ (mm/s)
PA2	2.7	4.9	3.3
PA3	8.3	15.6	0.8
PA4	5.4	4.4	6.4
	5.5 (3.0)	8.3 (6.0)	3.5 (3.0)
PB2	0.85	0.39	0.25
PB3	-	-	-
PB4	0.94	1.17	0.32
	0.90 (0.06)	0.78 (0.55)	0.28 (0.05)
PC2	0.37	0.41	0.65
PC3	0.38	0.53	0.23
PC4	1.69	6.51	0.43
	0.81 (0.8)	2.48 (3.5)	0.44 (0.20)
Beam	$V_{SG12}$ (m/s)	$V_{SG23}$ (m/s)	$V_{SG34}$ (m/s)
PA7	20.5	20.2	**20.48**
PA8	13.6	20.4	20.41
PA9	13.6	20.4	20.41
	15.9 (4)	20.4 (0.1)	20.43 (0.04)
PB7	10.2	8.2	1.5
PB8	10.2	13.6	3.1
PB9	-	-	-
	10.2 (0)	10.9 (3.8)	2.3 (1.2)
PC7	2.2	3.1	6.8
PC8	4.1	1.1	6.8
PC9	10.3	0.85	3.1
	5.5 (4.3)	1.7 (1.2)	5.6 (2.1)

**Table 7:** Measured crack velocities using the strain gages for the loading rate at 1.77 m/s (PX12, PX13 and PX14) and 2.66 m/s (PX17, PX18 and PX19), the corresponding mean and standard deviations are given in the fourth row of each concrete type. The maximum velocities in each group are marked as bold numbers.

Beam	$V_{SG12}$ (m/s)	$V_{SG23}$ (m/s)	$V_{SG34}$ (m/s)
PA12	-	-	-
PA13	304	**386**	121
PA14	266	327	94
	285 (27)	357 (42)	108 (19)
PB12	425	472	283
PB13	193	193	304
PB14	472	**472**	327
	363 (149)	379 (22)	305 (22)
PC12	386	304	266
PC13	**630**	333	125
PC14	425	327	147
	480 (131)	321 (16)	179 (76)
PA17	262	115	58
PA18	**1063**	354	150
PA19	354	283	104
	559 (438)	251 (123)	104 (46)
PB17	**708**	425	75
PB18	425	708	531
PB19	607	531	425
	580 (144)	555 (143)	344 (239)
PC17	250	**1417**	137
PC18	202	708	69
PC19	531	185	147
	328 (118)	770 (618)	117 (43)

**Table 8:** Measured crack velocities along the first 51 mm of crack extension, using the technique of DIC for the beams impacted at 2.66 m/s.

Beam	$V_{1}$ (m/s)	$V_{2}$ (m/s)	$V_{3}$ (m/s)
PA15	617	461	377
PB15	654	503	409
PC15	944	702	311

**Table 9:** Numerical results on the crack velocity (m/s) for the loading rate of 1.77 m/s (PX12, PX13 and PX14) and 2.66 m/s (PX17, PX18 and PX19), the mean values with the standard deviation in the parentheses are given in the fourth row of each concrete type. The maximum values in each group are marked as bold numbers.

Beam	$V_{12}$	$V_{23}$	$V_{34}$	$V_{45}$	$V_{56}$	$V_{67}$	$V_{78}$	$V_{89}$	$V_{9,10}$
PA12	224	264	260	254	223	197	129	86	54
PA13	252	296	**308**	298	331	242	172	113	72
PA14	222	254	260	245	210	192	132	83	66
	229 (20)	271 (22)	276 (27)	266 (28)	255 (66)	211 (28)	144 (24)	94 (16)	64 (9)
PB12	217	**289**	285	282	245	197	141	80	14
PB13	153	215	206	174	144	127	103	82	56
PB14	206	278	260	275	233	180	126	76	17
	192 (34)	261 (40)	251 (40)	244 (61)	207 (55)	168 (36)	123 (20)	79 (3)	29 (23)
PC12	583	651	667	694	436	409	256	150	89
PC13	718	725	667	**737**	476	425	254	149	89
PC14	313	440	482	600	359	347	264	144	87
	538 (207)	605 (148)	605 (107)	677 (71)	424 (59)	394 (34)	258 (5)	148 (3)	88 (1)
PA17	284	379	372	353	326	271	206	143	84
PA18	441	543	**589**	496	388	270	219	158	116
PA19	282	388	377	347	306	273	207	143	82
	336 (91)	436 (92)	446 (124)	399 (84)	340 (42)	271 (2)	211 (7)	148 (8)	94 (19)
PB17	206	347	359	326	274	240	184	131	78
PB18	237	366	**401**	342	293	237	202	139	93
PB19	195	311	316	288	258	233	176	120	75
	213 (22)	341 (28)	359 (42)	318 (22)	275 (18)	237 (3)	187 (13)	130 (8)	82 (9)
PC17	921	963	916	992	591	529	408	176	82
PC18	473	651	765	958	521	463	366	210	79
PC19	868	980	**1016**	**1016**	567	534	396	170	83
	754 (244)	865 (185)	899 (127)	989 (29)	560 (36)	509 (40)	390 (22)	186 (22)	81 (2)
